# Decoding unconstrained arm movements in primates using high-density electrocorticography signals for brain-machine interface use

**DOI:** 10.1038/s41598-018-28940-7

**Published:** 2018-07-12

**Authors:** Kejia Hu, Mohsen Jamali, Ziev B. Moses, Carlos A. Ortega, Gabriel N. Friedman, Wendong Xu, Ziv M. Williams

**Affiliations:** 1 Department of Neurosurgery, Massachusetts General Hospital, Harvard Medical School, Boston, MA USA; 2 Department of Hand Surgery, Huashan Hospital, Fudan University, Shanghai, China; 3 Department of Functional Neurosurgery, Ruijin Hospital, Shanghai Jiao Tong University School of Medicine, Shanghai, China; 4 Harvard-MIT Health Sciences and Technology, Cambridge, MA USA; 5 Harvard Medical School Program in Neuroscience, Boston, MA USA; 6 Behavioral Neuroscience Program, Northeastern University, Boston, MA USA; 7 Department of Neurosurgery, Brigham and Women’s Hospital, Harvard Medical School, Boston, MA USA

## Abstract

Motor deficit is among the most debilitating aspects of injury to the central nervous system. Despite ongoing progress in brain-machine interface (BMI) development and in the functional electrical stimulation of muscles and nerves, little is understood about how neural signals in the brain may be used to potentially control movement in one’s own unconstrained paralyzed limb. We recorded from high-density electrocorticography (ECoG) electrode arrays in the ventral premotor cortex (PMv) of a rhesus macaque and used real-time motion tracking techniques to correlate spatial-temporal changes in neural activity with arm movements made towards objects in three-dimensional space at millisecond precision. We found that neural activity from a small number of electrodes within the PMv can be used to accurately predict reach-return movement onset and directionality. Also, whereas higher gamma frequency field activity was more predictive about movement direction during performance, mid-band (beta and low gamma) activity was more predictive of movement prior to onset. We speculate these dual spatiotemporal signals may be used to optimize both planning and execution of movement during natural reaching, with prospective relevance to the future development of neural prosthetics aimed at restoring motor control over one’s own paralyzed limb.

## Introduction

Motor paralysis can be secondary to a disruption in the neural pathways between the brain and muscle without disrupting normal cognitive ability. Indeed many patients suffer from diseases, such as spinal cord injury, amyotrophic lateral sclerosis and cerebral palsy, but retain motor cortical circuitries necessary for planning and orchestrating movement^1,2^. Brain-machine interface (BMI) techniques can provide an indirect bridge between the brain and intact limbs and/or an external prosthetic device. By partially restoring lost motor function, BMIs may improve a patients’ ability to directly interact with their environment and provide a higher quality of life^3,4^.

Most previous BMI approaches have focused on the primary motor cortex (M1) as an area of brain signals for recording neural activity during movement, at it has been found to be directly relevant to movement execution and motor imagery^5–8^. BMIs that use signals recorded in M1 have yielded promising results for the control of robotic arms or even in patients’ own paralyzed limbs through functional electrical stimulation^9–12^. Unconstrained functional movements are involved in higher level cognitive aspects of motor control such as decision making, movement selection, and planning, and require complex interactions between multiple sensory, cognitive, and motor areas. Comparatively, the premotor cortex (PMC) may be an alternative cortical area of particular interest^13^. The PMC receives input containing sensory and volitional information from the prefrontal cortex (PFC) and posterior parietal cortex, and it produces output that goes to M1, which in turn sends motor commands for execution^14^.

As a specialized subarea of the PMC, the ventral premotor cortex (PMv) has been shown to relate to both motor output and cognition, including the cognitive functions of motor planning, spatial perception, and action organization^15^. Neurons recorded from monkey PMv contain representations of spatial goal-directed wrist movements^16^ and play crucial roles in transforming the three-dimensional visual properties of grasping movements^17^. In a human fMRI experiment, activation of PMv areas was observed during actions involving the arm reaching to grasp^18^. Therefore, while the prospective use of the PMv for BMI control may be high^19,20^, little is understood about how brain signals from the PMv alone may be used to control unconstrained reach-return arm movements in free-space. In particular, compared with neural spiking activity, whether or not this information can be accurately extracted from field potentials remains poorly understood.

Electrocorticography (ECoG) recordings have been widely used both in humans and non-human primates^21^, and provide a ‘midway level’ for abstracting brain signals between scalp EEG and intracranial single-neuron recordings. Compared with non-invasive EEG, ECoG recordings have higher spatial resolution and signal amplitude, broader recording bandwidth, and less interference from artifacts. On the other hand, ECoG signals also provide long-term stability and less invasive surgical procedures than surgeries to implant microelectrodes, which require penetrating the cortex to obtain single-unit neuron activity and local field potentials^22^. However, commonly used standard clinical ECoG grids, which help localize epilepsy foci intracranially, cover a relatively large area of cortex and each electrode is spaced a centimeter apart, thus making it challenging to spatially distinguish small nearby areas of neuronal activity^23^. Consequently, movements may be characterized with significant confusion and thus may be deemed inadequate for multi-degrees-of-freedoms (DOF) decoding, which is crucial to restoring functionally unconstrained movement.

High-density ECoG grids have been developed to improve clinical epilepsy localization precision, while also attempting to yield more accurate signals that better resolve extremity movements. High-density ECoG-based BMIs have been used to classify individual finger movements^24^, decode grasping force^25^, and provide robust control of a 3D cursor^26^. However, little is understood about how these signals may be used to potentially control unconstrained movements in free space, which needs movement planning, spatial perception, and a much higher level of multi-DOF decoding. There is also no information on how high-density ECoG signalsmay be potentially used in the PMv for BMI control.

Since patients’ paralyzed limbs cannot move, the awake-behaving non-human primate model is a good pre-clinical model for developing such a cortically-controlled movement paradigm. The purpose of this study was to explore the possibility of using high-density ECoG recordings from the PMv to identify functionally unconstrained reach-return arm movements performed by rhesus macaques. More specifically, we try to identify features in the ECoG signals that may help us determine the type and direction of movement.

## Results

**Figure 1:**
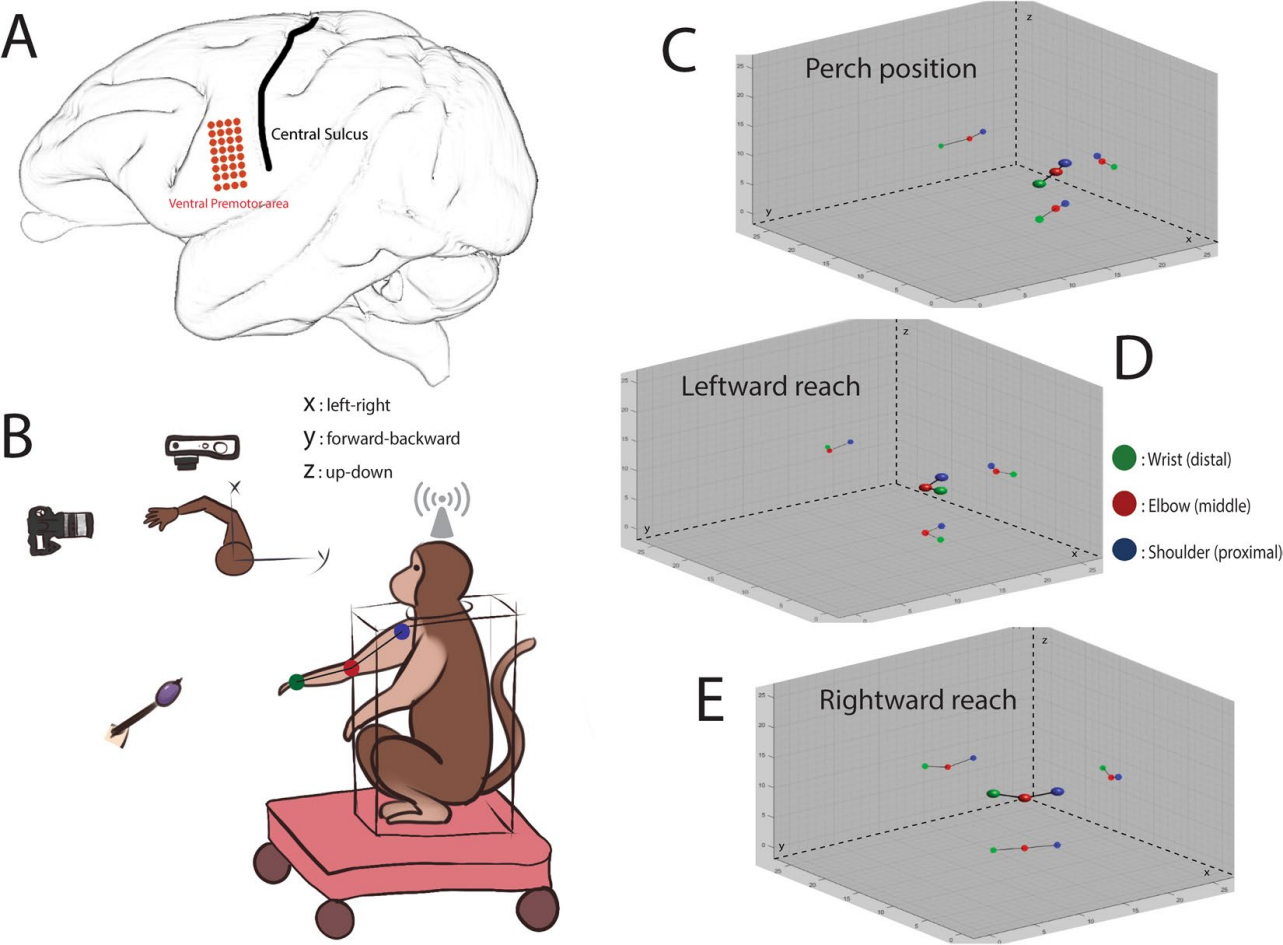
Overview of the experimental design and 3D rendered illustration of unconstrained arm movements. (**A**) The position of high-density ECoG array in the monkey’s left hemisphere. Red dots represent the 32 electrodes; the black line indicates the central sulcus (CS) of the left hemisphere. (**B**) Schematic diagram showing the experimental configuration of the reach-return task, where monkey was trained to reach for food offered by the experimenter in three-dimensional space without explicit cues; top-down view depicts the body-centered X-Y coordinates; the Z coordinate is perpendicular to the horizontal plane. Movement trajectories from XZ and YZ planes were recorded using two cameras, respectively. The wireless recording system received the ECoG signals from the monkey’s head stage, and transmitted these signals to the data acquisition system. **(C)** Starting position of the monkey’s right arm while perched. (**D**) Leftward reaching movement. (**E**) Rightward reaching movement. xyz scale values are in inches. Colorful dots were used to distinguish the distal (wrist joint with green color), middle (elbow joint with red color), and proximal portions (shoulder joint represent by triceps with blue color) of the right upper limb of monkey. The lines connected dots represent the forearm and the upper arm abstractly.

Two rhesus macaques were trained to perform the functionally unconstrained reach-return arm movements, while high-density ECoG or local field potential (LFP) signals were recorded from the PMv area (Fig. 1, see Methods). Overall, the food reach-return accuracy of the two monkeys was 99.4%, the reach duration was 1050 ± 290 ms, and the return duration was 590 ± 200 ms. Through the time-frequency analysis of ECoG and LFP oscillations, the entire frequency spectrum was divided into three frequency bands according to the similar characteristic modulation during the movement tasks: (1) low-frequency band (less than 9 Hz); (2) intermediate-frequency band (9–40 Hz); and (3) high-frequency band (greater than 40 Hz).

**Figure 2:**
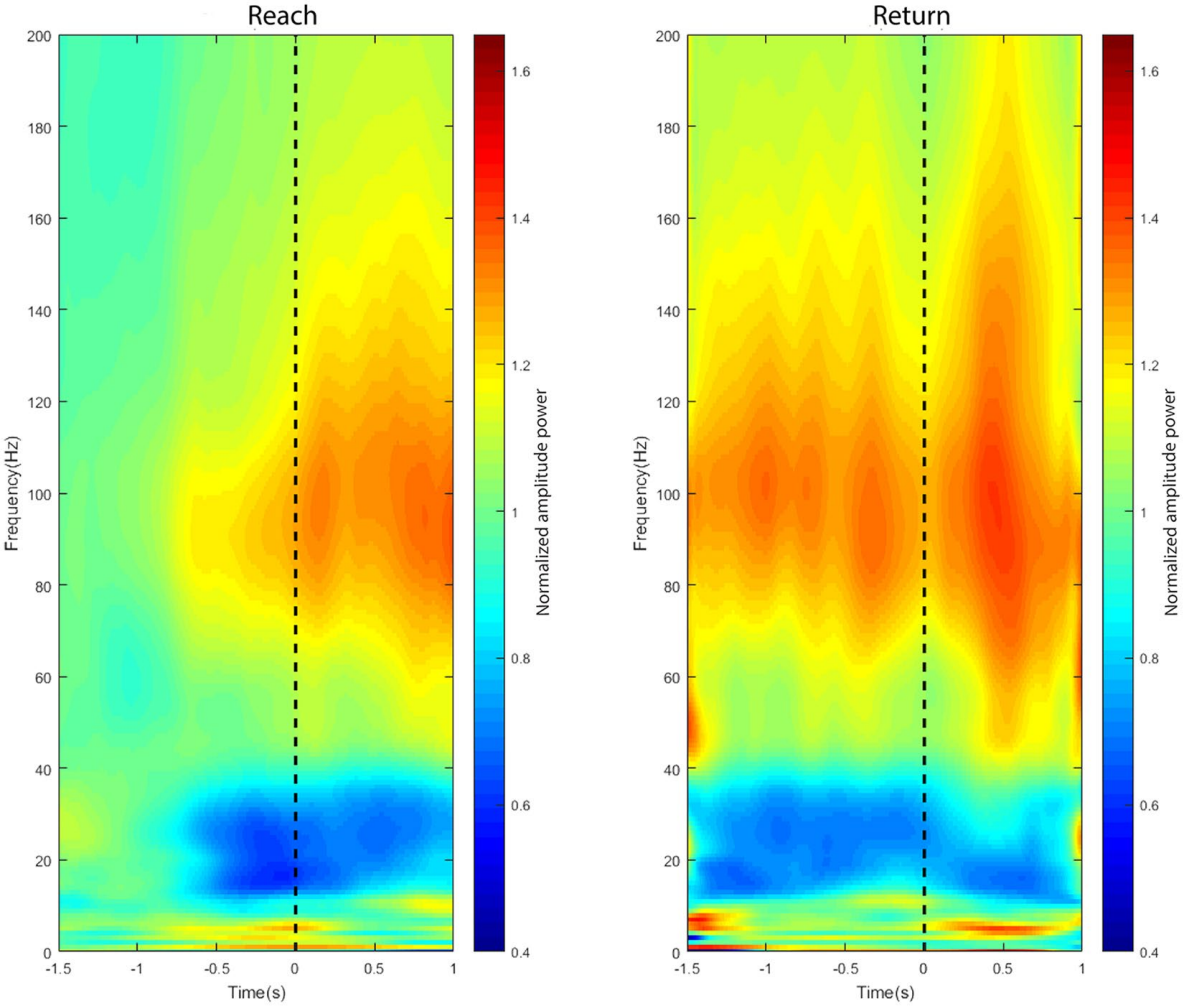
Spectrograms showing the time- and frequency-resolved amplitude spectra of reach-return movement from one representative PMv channel (Channel 10). The spectral power was averaged over all trials of reach-return movements for frequencies from 0–200 Hz and shown as a function of time relative to the event onset (dashed lines; left graph: onset of the reach movement, right graph: onset of the return movement).

Using the ECoG signal from monkey T, we observed a longer-lasting reduction of power amplitudes (event-related desynchronization, ERD) starting well before and ending after reach-return movements, in the intermediate-frequency band range (9–40 Hz). In a broad band of high-frequency signals from 40 Hz up to 200 Hz, a consistent amplitude increase (event-related synchronization, ERS) was observed before the movement onset during the movement period (Fig. 2).

**Figure 3:**
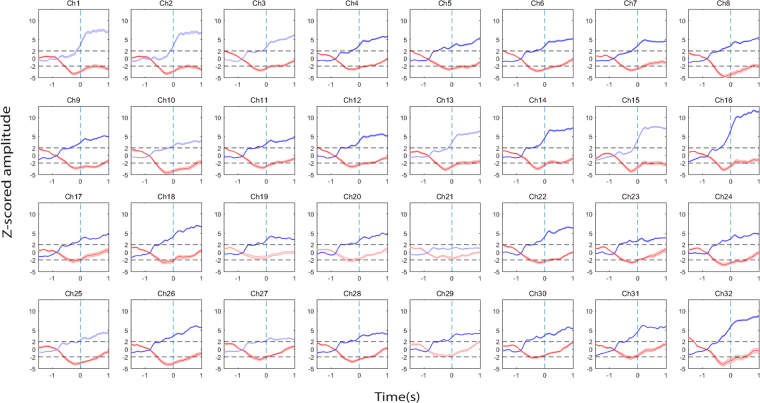
The relative power change in intermediate-frequency and high-frequency bands when compared with the baseline during reach-return movement for all 32 electrodes. For each channel a normalized (i.e., Z-scored) power change relative to the baseline power is plotted as a function of time. The dotted lines represent power changes equal to two standard deviations (2σ) away from that of the mean baseline value. Red: intermediate-frequency band; Blue: high-frequency band. Note that active channels for each frequency bands were highlighted using darker colors.

Next, we identified active channels as those exhibiting significant power changes during pre-movement or movement periods (Fig. 3). Here, we find that the average detectable times before movement onset are 531.68 ± 55.15 ms for the intermediate-frequency band and 460 ± 134.89 ms for the high-frequency band. Thus, the intermediate-frequency band power changes can be detected significantly earlier than the high-frequency band (*P* = 0.012).

**Figure 4:**
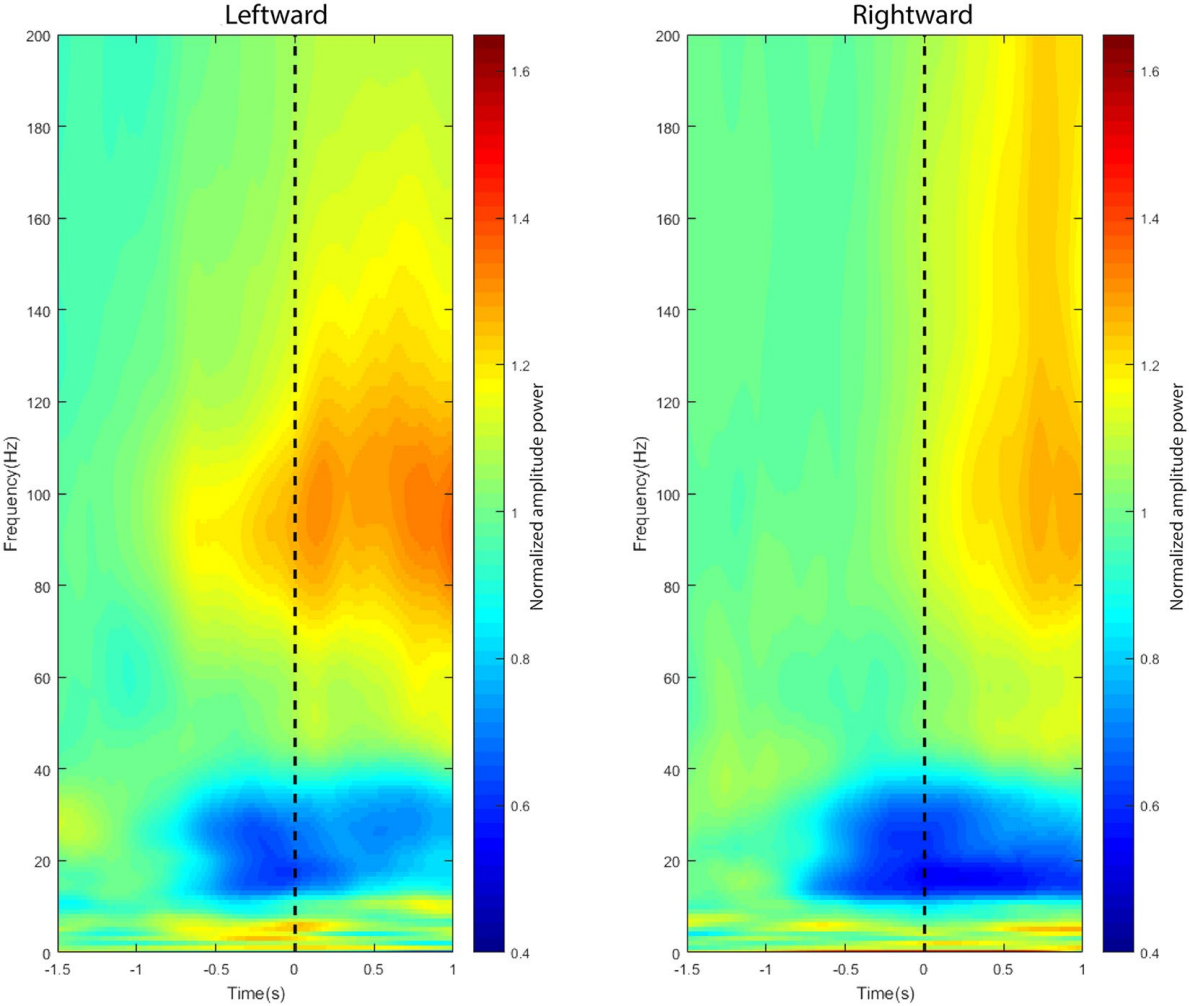
Spectrograms showing the time- and frequency-resolved amplitude spectra between leftward and rightward reach movement from one representative channel from PMv (Channel 10). The spectral power was averaged over all trials of left/right reach movements for frequencies from 0–200 Hz and shown as a function of time relative to the onset of movements (dashed lines; Left column: Leftward reach movement, Right column: Rightward reach movement).

Lastly, when we further separated movements into left and right reach directions (Fig. 4), we observed the same reduction of power amplitudes in the intermediate-frequency band and increase of power amplitudes in the high-frequency band for both directions of reach movement.

Regretfully, in this study only monkey T had been implanted with the high-density ECoG array. Therefore, we validated our finding in the same PMv area of monkey P, using the LFP signals from a floating microelectrode array (FMA) recording. We found consistent and similar spectral power changes with ECoG signals in the intermediate and high frequency bands (Fig. S1).

### Prediction of reach movement onset and left-right directionality

**Figure 5:**
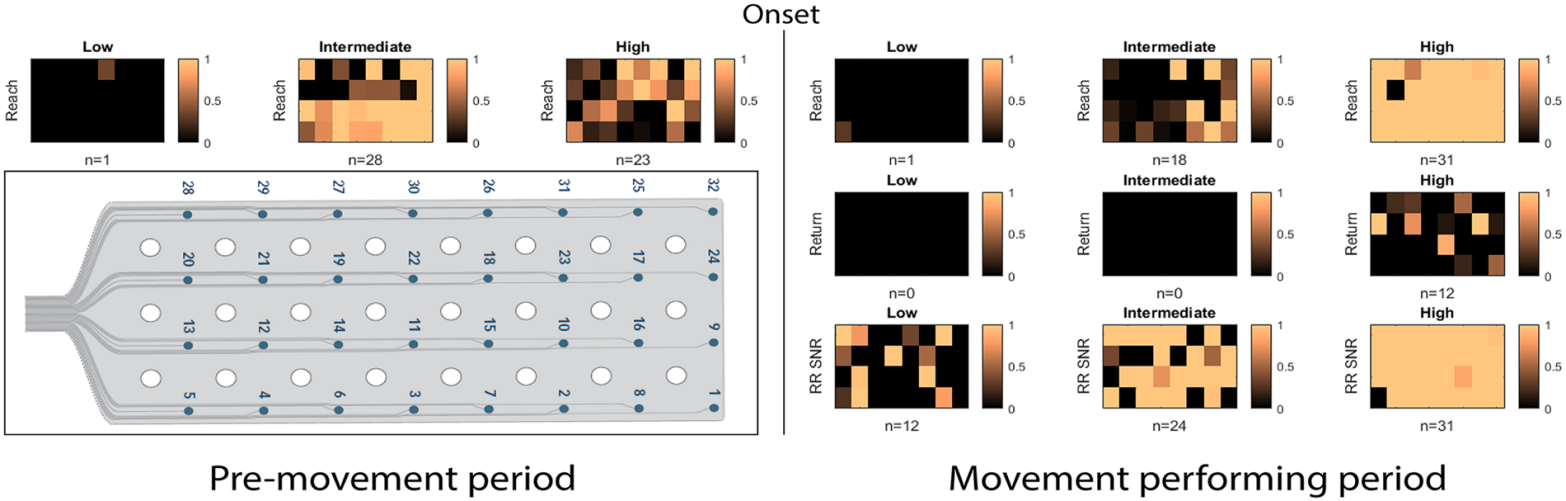
The layout of active channel patterns across all 32 recording electrodes during reach-return movements. Active channels corresponding to low-, intermediate-, and high-frequency bands before (left panels) and after (right panels) the onset of the movement (middle line). Channels are arranged to match the topographic map of ECoG electrodes (dorsal-to-ventral from left-to-right) shown at the bottom-left. The copper color bar 0-1 represents how strongly the relative power deviates from the baseline activity; 0 correspond to |Z-Score| of 2 whereas 1 represents |Z-score| ≥3. The number of active channels is reported below each panel. (Upper row: Reach, Middle row: Return, Lower row: Reach-Return movement SNR).

Here, when comparing the active channels using the ECoG signal from monkey T, we find that intermediate-frequency band neural activity from 28 of 32 channels and high-frequency band neural activity from 23 of 32 electrodes within the PMv could be used to accurately predict reach movement onset during the pre-movement period (Fig. 5).

**Figure 6:**
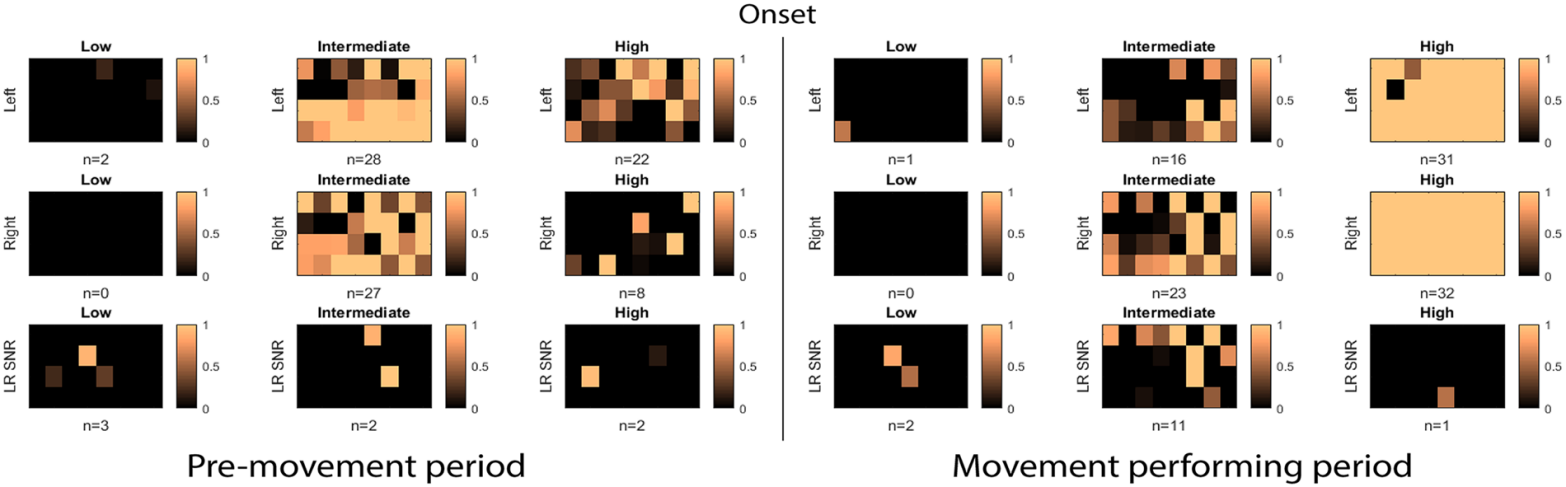
The layout of active channel patterns across all 32 recording electrodes during leftward and rightward reach movement. Active channels corresponding to low-, intermediate-, and high-frequency bands before (left panels) and after (right panels) the onset of the movement (middle line). Channels are arranged to match the topographic map of ECoG electrodes (dorsal-to-ventral from left-to-right) shown at the bottom-left of Fig. 5. The copper color bar 0-1 represents how strongly the relative power deviates from the baseline activity; 0 correspond to |Z-Score| of 2 whereas 1 represents |Z-score| ≥3. The number of active channels is reported below each panel. (Upper row: Leftward, Middle row: Rightward, Lower row: Left-Right state SNR).

**Figure 7:**
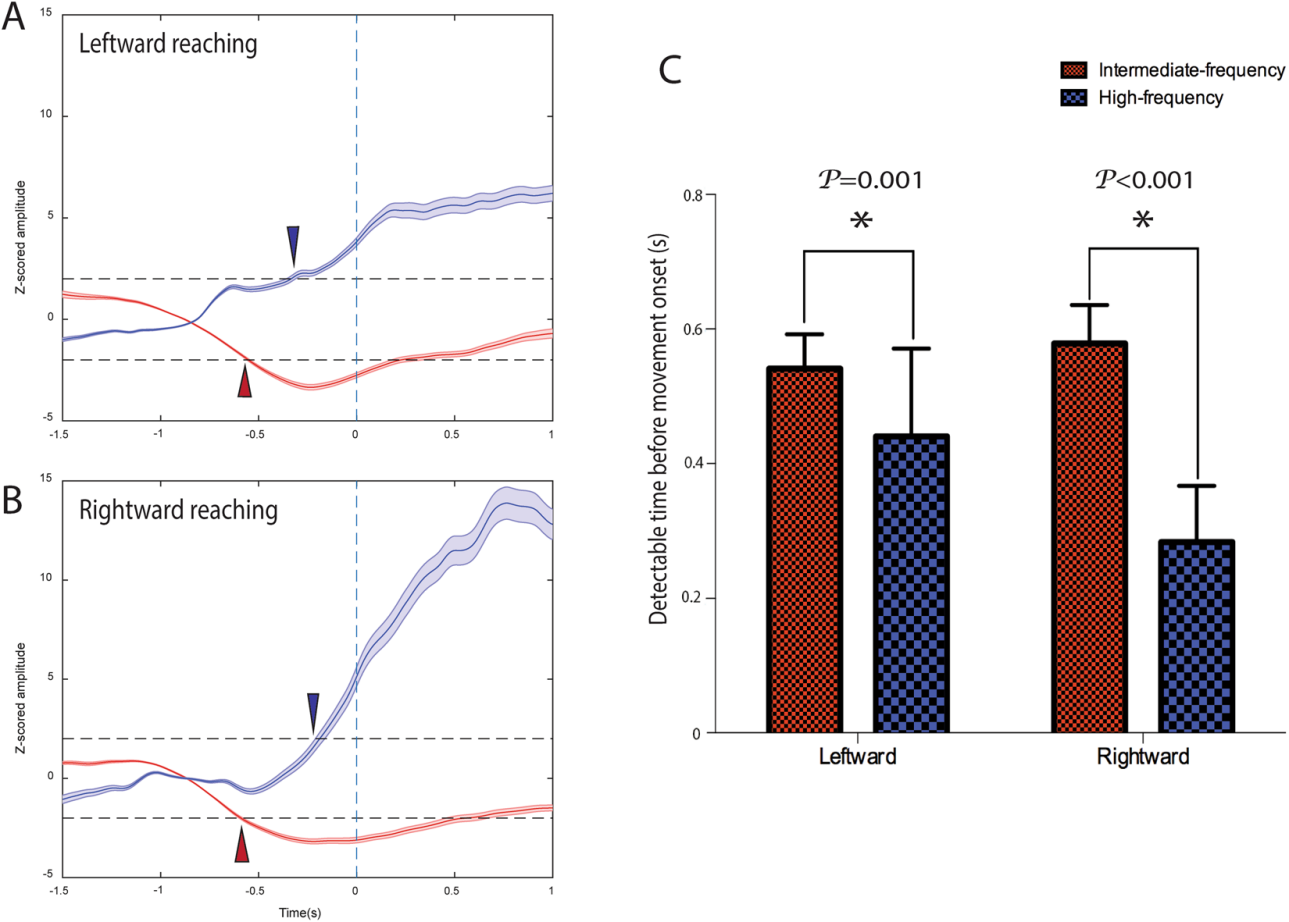
The average power change and the average detectable time of all active channels for leftward and rightward reach movement. The average normalized (i.e., Z-scored) power change relative to the baseline power across all active channels is plotted as a function of time for leftward (**A**) and rightward (**B**) reach movements. Bar graphs in (**C**) show the average time before the movement onset at which the power significantly differ from the baseline. Red: intermediate-frequency band; Blue: high-frequency band.

When comparing left-right directionality, intermediate-frequency bands continue to remain more predictive. In both left and right directions, intermediate-frequency bands had more active channels (Fig. 6) and significantly earlier detectable times than high-frequency bands (Fig. 7).

### Movement preforming representation

During movement execution, the high-frequency band was more informative of the planned movement, with 31 channels becoming active during the reach component and 12 channels during the return, compared with only 18 and 0 active channels when using the intermediate-frequency band, respectively (Fig. 5). Similar results were observed when directionality of the movements was taken into account (Fig. 6), with 31 channels becoming active during leftward movement and 32 during rightward movement in the high-frequency band. In both directions the high-frequency band resulted in significantly more active channels than when the intermediate-frequency band was used (left, 16 channels; right, 23 channels).

### Movement differentiation by SNR

Next, we asked how differentiable ECoG activity is for unconstrained: (1) reach movements between left and right directions; (2) the reach and return movements during movement execution.

During the pre-movement period, both intermediate-frequency and high-frequency bands had only two active channels which revealed significant SNR between left and right directions (Fig. 6). However, when analyzing all active channels, no significant difference (P = 0.390) was found in the mean SNR when comparing the intermediate frequency band of 3.014 ± 0.195 with the high-frequency band of 2.545 ± 0.578.

During the movement period, there were many more active channels of left-right SNR in the intermediate frequency band. The mean SNR of all active channels during this period was 3.141 ± 1.043, which is significantly higher than that found when using the high-frequency band (P = 0.046). In contrast, the SNR of reach and return periods had more active channels in the high-frequency band and significantly higher SNR (31 active channels, 6.464 ± 2.392) than in the intermediate-frequency band (24 active channels, 3.996 ± 1.049) (P < 0.001) (Fig. 6).

### Decoding results

We used a linear discriminant analysis (see Methods) to quantify the degree to which different components of ECoG/LFP signals were informative of the monkey’s movement type (reach vs. return) as well as the directionality of the reach movements (left vs. right). We were able to accurately and informatively decode movement related behaviors from the PMv area including all the channels from ECoG and LFP signals. Using the intermediate- and high- frequency bands, the reach and return movements could be identified accurately during movement execution, and the high-frequency band decoding accuracy is higher than that of the intermediate-frequency bands (Table 1).

**Table 1:** The accuracy of classification of reach and return movement (%).

Reach vs. Return	Signals	(Movement Onset to 500 ms)	
Frequency Bands		Intermediate	High
Monkey T	HD-ECoG	93.52	97.84
Monkey P	LFP	90.91	97.87

In monkey T with high-density ECoG signal, the decoding performance using intermediate- and high-frequency bands exceed the chance level to identify the direction of the reach movement during the pre-movement and movement execution periods, and the high-frequency region is slightly more informative than the mid-range frequency (Table 2). In contrast, the prediction of movement’s laterality in monkey P using LFP signal is less accurate.

**Table 2:** The accuracy of prediction and classification of Left and right reach movement laterality (%).

Left vs. Right Reach	Signals	Before (−500ms to 0 ms)		Movement Onset	After (0 ms to 500 ms)	
Frequency Bands		Intermediate	High		Intermediate	High
Monkey T	HD-ECoG	57.20	62.29		55.23	63.27
Monkey P	LFP	53.41	56.53		51.96	*Chance Level*

## Discussion

Our results suggest that using high-density ECoG-based wireless recording from the non-human primate PMv could provide a range of signal components for decoding both the movement state and laterality of functionally unconstrained naturalistic reach-return arm movements. On a coarse scale, intermediate and high-frequency components of EcoG signals seem to play distinctive roles during movement. While the power in the intermediate-frequency bands provided most of the information for predicting reach-return movement onset, it was power in the high-frequency bands (in particular the “high-gamma” band) that carried more information about reach-return movement execution. While limited by the use of an individual primate, we believe that this data provides important technical and conceptual advancements into the prospective use of the PMv for BMI use.

Achieving volitional unconstrained functional movement has been an important goal in the field of BMI development, as BMI control should be self-initiated and work in natural conditions. Goal-directed reaching movement has been widely used to study upper extremity motor control for developing BMI. Several studies have used compliant planar paths to simplify the kinematics and dynamics of arm movement when motion is constrained by external contact (i.e. two-dimensional movement)^27–31^, which facilitates movement selection and planning. Also, for monkeys, even simple tasks usually rely on very specific movements and over-training may then contribute to diminished differences in cortical representation.

Natural volitional movements, however, are three-dimensional and unconstrained. When subjects are not required to perform a straight movement or follow a specific trajectory, the spatiotemporal characteristics, control strategies and execution of the unconstrained movements become fundamentally different^32^. For a BMI system to have wide-ranging use in humans, it is necessary to train the decoding algorithm without large constraints such as those imposed when subjects just perform a specific motor task (e.g. moving a joystick) or simply 2-D movement (e.g. controlling a cursor on the screen), both of which can only achieve very limited functionality. Our results suggest a range of possible ECoG signal components from the PMv area that can be used for decoding functional unconstrained naturalistic reach-return arm movements, which may be of particular value in advancing the field of BMI development towards designs aimed at self-sufficiency for paralyzed patients.

ECoG signals have emerged as a potential control for BMI applications and they stand to gain wider adoption due to their unique ability to balance signal quality with implant invasiveness. Standard ECoG grids have been used in epilepsy patients to decode kinematics of arm movements in 3D space and classify movement and rest^33^. Our study has demonstrated that using high-density ECoG signals from a comparatively small brain area can accurately represent movement intention and execution. Control analysis also demonstrated that the decoding advantage of high-density over standard ECoG grids is manifest in the improved decoding accuracy^34^. The advantage is realized not by having a larger number of channels or covering a larger brain area but may be due to the higher electrode density and enhanced signal fidelity, which directly increases the probability that electrodes lie closer to or are directly over cortical generators of movement^35^. This hypothesis was supported by our analyses which showed that decoding performance can be significantly affected by electrode channel choices. Therefore, the development of computationally efficient algorithms for channel selection will also be an important issue for real-time applications in our future work.

Neuronal event-related oscillations that exist in the brain correspond to a wide range of frequencies and are usually categorized into five frequency bands: delta (1–3 Hz), theta (4–8 Hz), alpha (9–12 Hz), beta (12–30 Hz), and gamma (>30 Hz). These different frequencies are thought to reflect different sensorimotor or cognitive cortical processing^36^. Aforementioned ECoG studies mapping human sensorimotor cortex have shown that higher-frequency power amplitudes typically increase in association with actual or imagined movements, whereas the spectral power of lower frequency bands typically decrease in amplitude^37–40^.

In our intermediate-frequency band, the ERD components were mostly consistent with alpha and beta bands. Compared with data that alpha-band oscillations are related to working memory and short-term memory retention^41^, the functional significance of beta-band oscillations at present seems to be less known^42^. In the motor system, although beta bands have been classically understood as signals related to the maintenance of the current motor set at rest, recent theories have proposed that beta bands may involve an active process that promotes the existing motor set while considering neuronal processing of new movements^43,44^. Several studies have shown that beta bands in motor and premotor cortex can change depending on the expectancy of a forthcoming event. Using MEG, Donner *et al*.^45^ showed that choice-predictive activity changes of beta bands reflect a decision about an upcoming action already several seconds before it is executed while people watch a stimulus in a perceptual detection task. Rubbino *et al*.^46^ found when monkeys performed point-to-point instructed-delay reaching movements, the power of beta oscillation from LFP signals is enhanced around the visual stimulus cue onset and attenuated around the movement onset. It is not surprising, therefore, that in our study, the intermediate-frequency bands were more predictive of movement onset (more informative and earlier) compared with high-frequency bands.

ECoG recording allows for the direct recording of brain activity and with its high signal-to-noise ratio, it is particularly suited for the examination of higher gamma-band oscillation activity above 30 Hz. Gama-band oscillations are thought to play a crucial role in information processing, perceptual formation and object representation in cortical networks^47^. It is worth mentioning that the biggest power change of our high-frequency bands was above 80 Hz, the so called “high-gamma” band (80–200 Hz), which has been consistently observed in several cortical areas, suggesting independent functions and mechanisms^47,48^. High gamma-band synchronization in the sensorimotor cortex has been further studied using ECoG-signals in epilepsy patients. Pfurtscheller *et al*.^38^ showed self-placed movement in humans was induced by gamma ERS in the 60–90 Hz frequency band; Miller *et al*.^49^ found a spatially focal increase in power in a broad high-frequency band (76–100 Hz) during movement compared with rest. In our study, higher gamma-band oscillations displayed a sustained response and increased even prior to the onset of the movement to its cessation, which may reflect its unique response properties in the PMv.

Determining a person’s intent from brain signals, the where and when of movement, is a crucial component of BMI implementation^50–52^. Since paralyzed patients cannot move, to elicit specific patterns of neural activity signifying movement onset, patients are often asked to think about moving their arms or to have them observe an effector as it moves under computer control and imagine that they are moving it in the same way^5,53^. Lebedev *et al*.^54^ found directional selectivity in fast oscillations from premotor cortex can reflect specific aspects of an intended action. Interestingly, epochs of high attention to motor performance have been found to be associated with increases of synchrony between neurons. However, since a change of visual stimulus immediately precedes reach movement onset, this raises the possibility that the spectral power change may reflect the visual event changes instead of the reach onset^55^. Watanabe *et al*.^56^ also demonstrated beta oscillations from ECoG signals that were not strictly phase-locked to any of EMG onsets of muscle contractions but related more so to the attentive state and external cues.

In our experiment, we excluded the possibility of visual stimulation by using self-paced reach-return movement in the awake-behaving monkey, and found that the ECoG spectrum power changes were time-locked to the movement onset. By relying on internal choices for each trial in our study, the timing of the movement sequence was self-paced and the onset of movement was made by the subject. Therefore, we decoded signals generated by the subjects’ own intentions and actions, rather than by external commands.

The execution onset was observed in active channels approximately 0.5 s before movement initiation through a decrease in the spectral power in the intermediate-frequency bands while the power in the high-frequency (especially in higher-gamma) bands increased as the state transitioned from reach-return movement intention to overt movement execution. These results corroborate that the ECoG spectral change in the PMv is a robust indicator for movement prediction and can be used for BMI control.

Another important step is to examine whether ECoG signals from PMv can hold information about the status and directionality of movement. During movement execution, differentiating limb movements is critical and fundamental for BMI design. High-frequency bands showed better decoding performance than intermediate-frequency bands in separating reach and return movement components. Interestingly, intermediate-frequency bands were more informative at distinguishing left and right directionality during reaching execution.

There has only been scarce evidence that ECoG signals can give information about direction prior to movement. Wang *et al*.^57^ demonstrated that the onset of intended 2-D cursor movements and their direction could be detected using standard ECoG signals in intractable epilepsy patients, but not all the experimental subjects achieved good performance. In our study, we identified a few channels with significant SNR changes that could distinguish movement directionality before movement onset. The addition of signals that increase the dimensionality of information for left and right movements may be essential for restoring unconstrained 3D movement in paralyzed patients.

Nakanishi *et al*.^58^ decoded three-dimensional arm trajectories based on standard ECoG signals recorded from sensorimotor cortex of epilepsy patients. They reported the low frequency beta band had the highest and the high frequency gamma (50~90 Hz) bands had relatively high values for arm trajectories prediction. Nakanishi *et al*.^59^ also using the same methods predicted fingertip motions, claimed ECoG signals from the upper part of sensorimotor cortex included information concerning finger motions enough to control neuroprosthesis. Shin *et al*.^60^ chronically implanted ECoG arrays over the left M1, which had a diameter of 1 mm and an inter-electrode distance of 3 mm center-to-center. They verified that ECoG signals are effective for predicting muscle activities in time varying series when performing sequential movements. Chen *et al*.^61^ implanted the ECoG electrode arrays (1 mm diameter electrodes with inter-electrode distances of 3.0 mm) on the gyrus between the CS and the arcuate sulcus (AS) in the M1 area of monkeys’ left hemisphere. They decoded 3D hand trajectories and showed that most effective electrodes were concentrated at the lateral areas and areas close to the CS, especially in the δ (1.5∼4 Hz) and high γ (90∼150 Hz) bands. Compared with studies above, our results were not completely consistent. However, our implant contained 0.3 mm diameter platinum electrodes with inter-electrode distances of 3.0 mm, and we uniquely implanted the grid in the monkey’s ventral premotor cortex. Our study also demonstrated the viability of a wireless headstage system in the acquisition and transmission of ECoG. Carmena *et al*.^62^ reported that neuronal activity recorded from M1 showed greater efficacy than that from dorsal premotor cortex, supplementary motor cortex, posterior parietal cortex, and primary somatosensory cortex, but notably didn’t compare with vPMC. In turn, we believe our results provide a potential supplementary signal source for brain-machine interfaces applications.

Our study also demonstrated the viability of a wireless headstage system in the acquisition and transmission of ECoG. Existing wired ECoG recording systems use cables connecting the electrodes placed on the cortex with an external apparatus. This approach requires multiple percutaneous connections, thus increasing the risk of bleeding and infection, and allows for only short-term recording while the animal is physically connected. The advantages of fully implantable wireless ECoG recording systems derive from the absence of connecting cables thereby improving safety, subject comfort, and recording longevity^63^.

Behavioral studies often require many trials as well as stability in animals’ performance. Movement artifact is a common type of signal contamination in tethered recordings. Wireless technology has been shown to minimize the conventional unavoidable artifacts observed during *in vivo* electrophysiological recordings^64,65^, remove potential distractions and mechanical disturbances from cables, and allow for animals to move quickly during the execution of the task^66^. These sources of noise were shown to be minimized in our wireless recording system.

Our assessment of decoding performances was based on offline analyses. Real-time closed-loop decoding based on these signals might achieve higher performance than demonstrated here. Our immediate future work will focus on developing a classifier that will enable us to perform the classification while the task is being executed. This will particularly benefit development of real-time BMI applications. As noted above, the present experiments were aimed at providing a conceptual advancement and proof-of-concept, but how it scales with more wide-ranging movements and animal participants will require further investigation.

## Conclusion

Collectively, our findings suggest that using wireless high-density ECoG recording from PMv provide a range of signal frequency bands that can be used to decode the state and onset of natural self-placed reach-return movements. We demonstrated the ability to predict the onset and laterality of reach-return movements in a non-human primate model. These ECoG signal components can serve as potential candidates for future use in an ECoG-based BMI technology that would allow for the neuronal control of unconstrained movements in paralyzed people.

## Methods

### Subjects and Materials

Two adult male Rhesus macaques (monkey T and P), aged eight and ten years old, were included in this study in accordance with our institutional IACUC guidelines and approved by the Massachusetts General Hospital Institutional Review Board. The monkeys were previously implanted with titanium head fixation posts for head stabilization. A 32-multichannel high-density ECoG electrode array (NeuroNexus, USA) was chronically implanted in the subdural space of monkey T’s left hemisphere covering the PMv (Fig. 1A). The implant contained 0.3 mm diameter platinum electrodes with inter-electrode distances of 3.0 mm. Two 16-contacts floating microelectrode arrays (FMAs) (NeuroNexus Technologies Inc., MI) were surgically implanted in areas of the PMv of Monkey P, to record LFP signals.

The correct placement of ECoG arrays and FMAs were confirmed using inspection of sulcal and gyral anatomy. Electrical cables leading from the ECoG and micro-electrodes array were connected to an interface (Omnetics, USA) affixed to the skull with titanium screws and dental cement.

### Behavioral and Neurophysiological Recordings

Next, to demonstrate that these techniques could be potentially used in unconstrained individuals, experiments were performed using a wireless headstage transmitter and data acquisition system (TBSI, USA) that fed neural signals into a customized multiacquisition processor system (Plexon, USA). ECoG signals were recorded at a sampling rate of 1KHz per channel. For FMAs, we confirmed that no single-units were present on LFP channels by thresholding and principal component analysis.

As detailed further below, the monkeys’ arm was unconstrained. Here, arm movements were captured using two video cameras placed in an orthogonal manner to provide three-dimensional trajectories. Offline analysis of movement was performed using customized software (Panlab, Harvard Apparatus, USA). The day prior to the experiment the monkeys’ right arms were painted using water-soluble dye to distinguish the distal (wrist joint with green color), middle (elbow joint with red color), and proximal portions (shoulder joint represent by triceps with blue color) of the upper limb. These different color circles represent three-dimensional markers for the motion capture system, which provided the needed fiducials to track the unconstrained limb.

### Experimental procedure

The monkeys were seated head-fixed in a custom primate restraint chair facing the experimenter (Fig. 1B). The animals could chew the apple pieces; however, they could not have other head movements during the experiments. The wireless headstage was connected and both video recording and neural signal acquisition were synchronized with the use of an analog signal trigger. The monkeys were trained to rest their right hands on a perch bolted to their chairs. Prior to initiating a movement to retrieve food items in free space, the monkeys’ non-acting hands were restrained on an arm rest with Velcro bands. After retrieving the food, the monkeys were free to consume the reward and a new trial was initiated with placement of their hands back on the perch. The monkeys were trained to only use the hand contralateral to the implanted hemisphere. A pseudo-randomized set of left and right locations was chosen prior to each experiment in order to ensure an adequate number of trials in each direction (Fig. 1C–E). The experiment was repeated over 10 days, with a minimum of 80 trials/day. The trial time was 5 s and the experimenter waited for the monkeys to finish the chewing before starting the next trial, which normally took 10–20 s, whereas the whole session took about 30 minutes. A small piece of apple was used for every trial, which was one of the two monkeys’ favorite food. The approximate size of each apple given was 0.5 cm*0.5 cm*0.5 cm. Trials were excluded from analysis if one of the following criteria was met: (1) The monkey reached inaccurately (e.g. missed the targets); (2) The monkey didn’t finish the reach-grasp-return period during the trial; (3) During the present trial, the monkey accidently chewed the residual food from prior trials.

### Data collection and analysis

The monkeys’ reach-return movements were self-paced, and the reach (including directionality) and return movements were identified on a trial-by-trial basis. The components of a trial were defined as follows: (1) reach onset as the time when the monkey’s hand left the perch which was identified by a pressure sensor; (2) return onset as the time when the monkey withdrew his hand from the food target, which was identified by looking back at the videos frame by frame and extracting the detailed frame time, with knowledge that the frame rate of the videos was 29.97 frames/second. Three periods which all consisted of intervals of 0.5 s were observed: (1) a baseline period (from 1.15 s to 0.65 s before movement onset; that is, the reference value that was used to calculate the relative power changes); (2) a pre-movement period (from 0.5 s to 0 s before movement onset); (3) a movement performing period (from 0 s to 0.5 s after movement onset).

All ECoG activity data was referenced to a common ground. The average voltage of each channel over the whole block was subtracted to prevent possible drift, and the signal from each channel was divided by its standard deviation over the entire block to normalize for systematic differences in amplitudes.

Then, a time-frequency analysis of the ECoG signals was performed. The power spectrum of the ECoG signals was analyzed for each channel and each type of movement. Discrete Meyer wavelet analysis was used to isolate frequency components of event-related spectral power changes and identify the characteristics of activities. Due to decreasing power with increasing frequencies, and in order to present spectral modulations over a large frequency range and to examine the large variations in spectral power, each frequency band was divided by the trial- and time-averaged amplitude value during the baseline period to account for the large variations in spectral power over different frequencies.

We identified three frequency power bands with characteristic modulation during the movement tasks: (1) a low-frequency band (less than 9 Hz); (2) an intermediate-frequency band (9–40 Hz); (3) a high-frequency band (greater than 40 Hz). These frequency bands are determined from the center frequency of the Meyer wavelet, decomposition scale, and sampling frequency.

For the comparison of time- and frequency-resolved ECoG amplitudes from the movement types and directions, we used the signal-to-noise ratio (SNR) to assess the strength of a specific signal relative to the noise caused by trial-to-trial variability when performing the task. The SNR is defined by the difference of the class means (as an estimate of the signal) divided by the average trial-by-trial fluctuations (as an estimate of noise);

1 $$SNR=\frac{|{\mu }_{1}-{\mu }_{2}|}{0.5\,\times ({\sigma }_{1}+{\sigma }_{2})}$$

where μ1 and μ2 are the means of the two classes or directions of movements; σ1 and σ2 are their standard deviations across trials.

A channel was considered active for a certain frequency band if the mean power during the pre-movement or movement period was significantly different than the baseline. The Kolmogorov-Smirnoff test was used to test whether the data were normally distributed. Normally distributed data was expressed as mean ± standard deviation, and skewed data was expressed as median (interquartile range). F-test was used for homogeneity of variance, independent samples t-test for equal variance, and non-parametric test was used for unequal variance. P < 0.05 was considered statistically significant. The detectable time is defined as the earliest time at which the power change was different from that of the baseline before movement onset.

The power changes of active channels were visualized with Z-score. More than two standard deviations (2σ) away from that of the mean baseline value was considered as statistically significant change, which is equal to |Z-score| (absolute value or modulus of Z-score) >2.

### Decoding Algorithm

A Fisher’s discriminant analysis was used to quantify the degree to which ECoG or LFP signal components were informative of the monkey’s movement type (reach vs. return) and its direction (left vs. right). We quantitatively measured the ratio of the variance in ECoG neuronal activity between the group options (reach vs. return/left vs. right reach) to the variance within the groups based on:

2 $${{{\rm{S}}}_{{\rm{w}}}}^{-1}{{\rm{S}}}_{{\rm{B}}}{\rm{v}}={\lambda }{\rm{v}}$$

whereby S_w_ and S_B_ are the within group scatter matrices and between group scatter matrices, respectively. The prediction vector v, corresponds to the largest eigenvalue of the matrix on the left-hand side of the equation. The prediction vector defines a projection of the recorded activity into a scalar unit that is then compared to a threshold, θ, and to predict the trial choice. For validation, we divided the data into a training set consisting of 75% of the trials and tested the accuracy of the prediction on the remaining 25% of trials. This operation was repeated 1000 times using a random sampling of the total trials.

## Electronic supplementary material

Supplementary Figure
