# Supplementary material for: Decoding unconstrained arm movements in primates using high-density electrocorticography signals for brain-machine interface use
Source: Sci Rep. 2018 Jul 12;8:10583. doi: 10.1038/s41598-018-28940-7 (PMC6043557; doi:10.1038/s41598-018-28940-7)
Supplement: Supplementary file 1 — Supplementary Figure [file 41598_2018_28940_MOESM1_ESM.pdf]

Supplementary Information

Decoding unconstrained arm movements in primates using high-density electrocorticography signals for brain-machine interface use

Kejia Hu[1,2,3]*, Mohsen Jamali[1], Ziev B. Moses[1,7], Carlos A. Ortega[6], Gabriel N. Friedman[1], Wendong Xu[2], Ziv M. Williams[1,4,5]*

[1]Department of Neurosurgery, Massachusetts General Hospital, Harvard Medical School, Boston MA, USA

[2]Department of Hand Surgery, Huashan Hospital, Fudan University, Shanghai, China

[3]Department of Functional Neurosurgery, Ruijin Hospital, Shanghai Jiao Tong University School of Medicine, Shanghai, China

[4]Harvard-MIT Health Sciences and Technology, Cambridge MA, USA

[5]Harvard Medical School Program in Neuroscience, Boston MA, USA

[6]Behavioral Neuroscience Program, Northeastern University, Boston MA, USA

[7]Department of Neurosurgery, Brigham and Women's Hospital, Harvard Medical School, Boston MA, USA

K.H, M.J and Z.B.M contributed equally to the work

Corresponding Authors: Kejia Hu, Email: dockejiahu@gmail.com;

& Ziv M. Williams, Email: zwilliams@mgh.harvard.edu

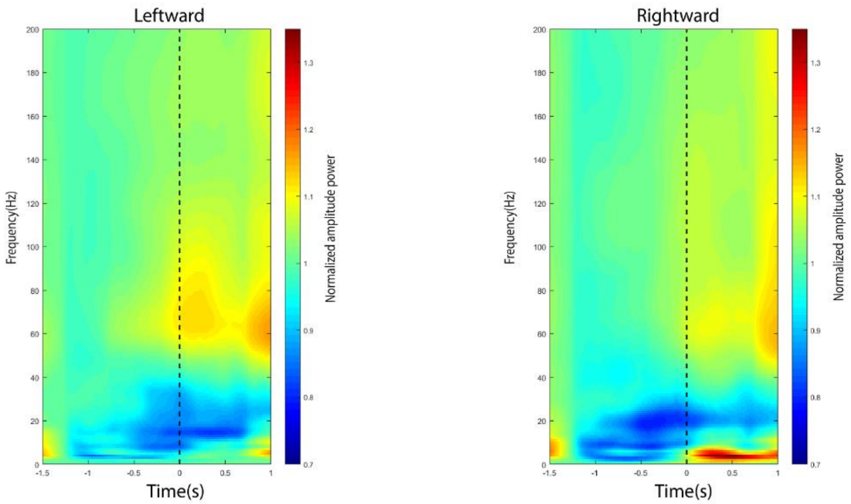

Figure S1. Spectrograms showing the time- and frequency-resolved amplitude spectra of leftward and rightward reach movement from one representative LFP channel (Channel 21) of monkey P. The spectral power was averaged over all trials of left/right reach movements for frequencies from 0–200 Hz and shown as a function of time relative to the onset of movements (dashed lines; Left column: Leftward reach movement, Right column: Rightward reach movement).
